# Patient Unpunctuality’s Effect on Appointment Scheduling: A Scenario-Based Analysis

**DOI:** 10.3390/healthcare11020231

**Published:** 2023-01-12

**Authors:** Ping-Shun Chen, Hsiu-Wen Chen, Marielle Donice M. Abiog, Roxanne Mae B. Guerrero, Christine Grace E. Latina

**Affiliations:** 1 Department of Industrial and Systems Engineering, Chung Yuan Christian University, Chung Li District, Taoyuan City 320314, Taiwan; 2 School of Industrial Engineering and Engineering Management, Mapúa University, Intramuros, Malina 1002, Philippines

**Keywords:** patient appointment scheduling problem, unpunctual patient, simulation, preempt policy, wait policy

## Abstract

This study examined patient unpunctuality’s effect on patient appointment scheduling in the ultrasound department of a hospital. The study created a simulation system incorporating the formulated F3 distribution to describe patient unpunctuality. After the simulation model passed verification and validation processes, what-if scenarios were conducted under two policies: The preempt policy and the wait policy. A comparison of the total cost of each policy showed that the preempt policy performed better than the wait policy in the presence of unpunctuality. The study used sensitivity analyses to identify the different effects of patient unpunctuality on the system. The weights of the cost coefficient of both radiological technician’s idle time and patient waiting time must be equal in order to achieve a lower cost. The patient’s inter-arrival time must be close to the average total time in the system to achieve lower costs. Moreover, utilization decreases as the patient’s inter-arrival increases. Therefore, the patient’s inter-arrival time should be higher than, but close to, the service time to ensure less radiological technician’s idle time and patient waiting time.

## 1. Introduction

Healthcare has grown into one of the major industries in the world, with the purpose of providing patients the best and most cost-effective care possible [1]. Today, healthcare services are working to be more patient-centered than provider-centered [2,3]. Scheduling practices in this sector differ in terms of the type of service offered, whether for an emergency, outpatient, inpatient, or specialty care. These scheduling methods appoint patients to various time slots through appointment scheduling policies [4,5,6].

In healthcare, appointment systems aim to bring balance between capacity and demand by improving operational efficiency and client satisfaction via decreasing the uncertainty in patient’s arrival and regulating patient demand [7]. Poor appointment policies may result in longer patient waiting times and low provider utilization [8,9,10]. As the need for healthcare services continues to grow, the problems that providers face are also increasing. Most clinics and hospitals want to minimize overtime and/or idle time of their resources while patients do not want to wait long hours to get their desired service [5]. This situation has attracted the attention of researchers. Furthermore, researchers have integrated appointment policies with patients’ behaviors (such as unpunctuality or no-show patients) for exploring the patient appointment scheduling policies [11,12].

Patient unpunctuality is generally defined as the difference between the time the patient arrived and the start of his or her scheduled appointment time, regardless of whether the patient arrives late or early for the appointment [13,14]. If the patient arrives before the appointment time, the early patient will wait until the appointment time to receive the service or receive the available service. This will depend on appointment policies. If a patient arrives after the appointment time, the doctor (i.e., the service provider) will be not sure whether to preempt the early patient or wait for the missing patient. This will also depend on appointment policies. For the preempt and wait policies, Samorani and Ganguly [15] created an analytical method to determine the intervals for when it is optimal to wait and when it is optimal to preempt. They assumed that appointment slots have the same length as the mean service time and are given to one patient only [16]; they also assumed that walk-ins are not allowed. Their analytical results show that preempting is not always the solution for unpunctuality, especially for a clinic with long consultation times. In fact, the service provider should wait for the missing patient even if there is another patient waiting.

Deceuninck et al. [17] extended Samorani and Ganguly’s [15] study to construct their own mathematical model for solving this dilemma. In their study, they considered stochastic consultation times and scheduled patients with increasing consultation time variability. Their finding shows that the doctor should stay idle and wait for the missing patient to arrive, supporting the results of Samorani and Ganguly [15]. Moreover, they found that Samorani and Ganguly’s [15] analytical method was only applicable for solving the wait–preempt dilemma when a medical practice faced a low overtime cost, a high probability of no-shows, and long consultation times.

In the current study, the researchers investigated a hospital’s ultrasound department that contained multiple scanning rooms. The service time for each patient varies as different body parts are examined. Furthermore, both outpatient and inpatient were considered and were randomly assigned in the rooms in the hospital. The researchers focused on testing different scenarios using system simulation incorporating two traditional policies—namely, preempt and wait policies—to determine how early, on-time, and late patients affect the performance of patient appointment scheduling.

The objective of this study is to investigate the impact of patient unpunctuality on the performance of patient appointment scheduling problems using system simulation. The research procedures of this study are as follows: (1) Create a model that represents the real system; (2) generate a probability distribution of patient behavior; (3) create wait and preempt policies for patient unpunctuality; (4) compare and choose the best among the created policies; and (5) use the chosen policy to conduct sensitivity analyses in order to determine the impact of patient unpunctuality on the performance of the system. This study can be used as a tool for hospital managers when making decisions regarding which sequential rule to implement. The study was conducted in an ultrasound department with many rooms, and only patients with appointments were included.

The next section reviews the relevant literature. Section 3 presents the research procedure. The results are presented in Section 4. Section 5 offers discussions and Section 6 presents our conclusions.

## 2. Literature Review

Previous researchers have scrutinized appointment scheduling in order to determine the optimal policy that is feasible to be implemented in healthcare systems. The first research study that focused on the patient appointment scheduling problem was Bailey’s rule [18,19], a traditional rule that appointed two patients in the first time slot. Individual patients were scheduled for the subsequent time slots at a fixed interval [7,13,18]. The majority of patient appointment scheduling policies created have been correlated with and assessed using Bailey’s rule. Patient appointment scheduling policies that have been shown to perform better than Bailey’s rule include increasing interval and clustering rules [13], which group patients with short patient appointment intervals in the first half of the session and those with long appointment intervals in second half of the session. This plateau dome policy [13,20] schedules the number of slots in the first and last slots of a time interval while patient appointments that have the same time slot are scheduled in the middle of a time interval using the dome rule [7]. According to this rule, appointment intervals increase in length toward the middle of the session and decrease toward the end of the session.

Several studies have assumed that appointed patients are punctual when practices formulate their appointment scheduling policies. In addition, several patient appointment scheduling policies consider constant patient arrival time at constant time intervals, such as individual-block/fixed-interval rule [7] and constant arrival policy [5]. Chen et al. [5] also considered constant patient arrival policy, plateau dome policy, and the mixed patient arrival policy. The latter policy includes two models (M1 and M2). In M1, more patients are booked in the first slot rather than the second slot; in M2, more patients are booked in the second slot rather than the first slot. Chen et al. [5] formulated a patient appointment scheduling policy that was called the three-section pattern arrival policy, which depended on the length of the patient interval. This policy divided the time interval into early morning, midday, and late afternoon. Long patient appointment intervals were scheduled for midday, while short patient appointment intervals were scheduled in the early morning and late afternoon. The dome rule [7], increasing interval, and clustering rule policies [13] were similar to the three-section pattern arrival policy [5] in terms of the patient appointment interval.

Patient unpunctuality negatively impacts the performance of patient appointment scheduling systems [13,21], although it is not an unusual event in real-life healthcare settings. Few patient appointment scheduling studies have considered patient unpunctuality using simulation optimization [13,15,17]. Previous researchers have shown that patients are likely to show up earlier than the patient scheduled before them, arriving on average 17 min early [15,16].

A few studies about patient unpunctuality are available in the literature [17,21,22,23,24,25,26,27]. Several published studies adopted data-fitting methods to determine appropriate probability distributions without considering patient arrival pattern characteristics, such as constant variability, skewness, and leptokurtic patterns [14,17,28,29,30,31]. Due to the complex data-fitting methods, they tend to assume that all patients are punctual. In order to fit the data with patient unpunctuality, Tai and Williams [31] formulated a hybrid distribution modeling technique called F3. In their model, they used an asymmetrical frequency probability distribution of data wherein they determined three modes and then grouped the original data into *n* datasets. They compared the F3 distribution with normal and Pearson VII distributions; based on the F3 distribution’s graphical probability plots, they confirmed that F3 has the most accurate fitting.

Although researchers have studied different characteristics of patient appointment scheduling problems, the majority of these studies have focused on the allocation of patients using a single-server or one doctor only [7,13,17,32]. Some existing multiple server and multistage studies did not tackle the effects of patient unpunctuality [33,34,35], but instead developed a scheduling approach to deal with the patient appointment scheduling problem [2,8,36]. Furthermore, researchers have applied mathematical models [37,38], system simulations [5,6,39,40], and heuristic or meta-heuristic algorithms [41,42,43] to solve patient appointment scheduling problems. In order to deal with a complex (i.e., random and uncertain) patient appointment system, most researchers have used system simulation as the methodology for solving patient appointment scheduling problems [5,6,39,40]. Therefore, this study uses system simulation to investigate the impact of patient unpunctuality in a multi-scanning room environment, which belongs to a multi-server system, thereby filling this research gap. This is the motivation of the study.

## 3. Methodology

### 3.1. Problem Statement

Patient unpunctuality is defined as the time when a patient arrives early or late for the scheduled appointment. Commonly, the hospital has multiple scanning rooms, thus multiple patients can be served simultaneously. Most rooms are multi-purpose in order to provide patients with any type of service necessary. The preempt or wait policy is applied when the scheduled patient is late or an early patient is present. This approach aims to ensure that the flow of service is not interrupted. However, patient unpunctuality interferes with the appointment scheduling policy.

The case hospital provided 6 months of data, including the patient type and gender, scanned body position, check-in time, appointment time, and start and end times of scanning and imaging. These data were based on the current appointment scheduling policy used by the case hospital. In this study, only patients with scheduled appointments were considered, including outpatients and inpatients. Outpatients are patients who receive service or treatment without staying in the hospital overnight. Inpatients are patients who receive service and treatment while staying in the hospital for a 2 days or weeks.

**Figure 1:**
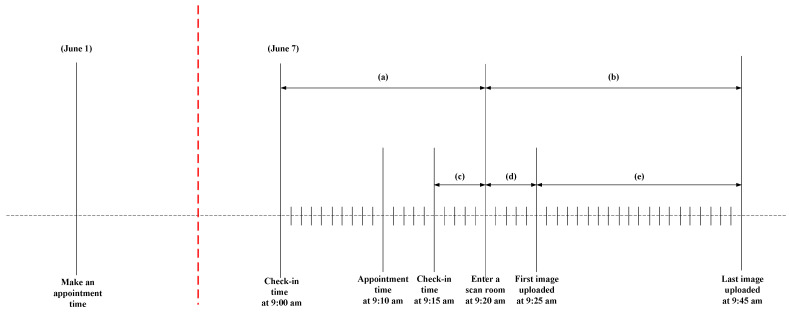
An example of the recorded time for a patient’s scanning appointment procedures at the case hospital.

The current appointment scheduling policy of the case hospital uses the constant arrival policy, wherein each patient is scheduled for 20 min of scan time. Outpatients and inpatients are scheduled for eight and six types of ultrasound scans, respectively. Figure 1 explains how the system works in the hospital. As observed in the figure, a patient goes to the hospital on 1 June to make an appointment for 9:10 am on 7 June. The patient returns to the hospital for the scheduled appointment on 7 June. Upon arrival, the check-in time of the patient is recorded; the check-in time can be earlier (i.e., 9:00 am) or later (i.e., 9:15 am) than the appointment time depending on the patient’s arrival. If the patient arrival time is 9:00 am (i.e., early patient) and the scanning room is available at 9:20 am, the total patient waiting time is denoted by (a), which is 20 min. If the patient arrival time is 9:15 am (i.e., late patient) and the scanning room is available at 9:20 am, the total patient waiting time is denoted by (c), which is 5 min. The patient’s scan time starts when the patient enters the scanning room at 9:20 am and continues until the final upload of the scan image is conducted at 9:45 am. Therefore, the total time of patients in the scanning room is denoted by (b), which is 25 min. The total service scan time includes the preparation time (i.e., 5 min denoted by (d)) and the service time (i.e., 20 min denoted by (e)).

### 3.2. Data Collection

Patient unpunctuality time, expressed in minutes, is the time between the check-in time and appointment time of outpatients and inpatients. Patient unpunctuality time was used for the data-fitting procedure to determine the probability distribution for unpunctuality of each type of patient. It was used as the basis to determine whether a patient is early, on time, or late. A patient with 0-min unpunctuality time is considered on time.

Six months of data include outpatients, inpatients, and emergent patients. Emergent patients were excluded since walk-ins without appointments were not considered in this study. Both outpatients and inpatients had outliers. Outliers that are three standard deviations from the mean were removed. Outpatients whose unpunctuality time was less than −50 min or more than 20 min were removed; inpatients whose unpunctuality time was less than −15 min or more than 10 min were also removed. Here, a patient with a −50-min unpunctuality time means that the patient arrived 50 min before the scheduled appointment time.

Service time is the difference between the first and last upload of the scanned image. Service times that took more than 1 day to finish were deleted since these data were incorrectly measured, which can cause abnormalities and inaccuracy in the system. Furthermore, service times that were less than 1 min or more than 40 min were deleted based on the definition of service time outliers. After deleting wrong data and outliers, the remaining data were used for the data-fitting procedure. The data-fitting results are introduced in the following section.

### 3.3. Data Fitting

All outpatient and inpatient data were fitted using the input analyzer toolbox of Arena simulation software (Rockwell Automation, Coraopolis, PA, USA). The results show that the best fit for outpatients is a normal distribution while for inpatients it is a beta distribution. However, both distributions failed in the Chi-square test as their *p*-values were less than 0.05. Tai and Williams’s F3 distribution model [31], the so-called hybrid distribution, was applied. The data were divided into subsets based on the results in fitting each subset to get a probability distribution.

Table 1, Table 2, Table 3 and Table 4 show the F3 distributions for early outpatients, late outpatients, early inpatients, and late inpatients, respectively. Based on the data-fitting results, the overall probability density function for early outpatients, late outpatients, early inpatients, and late inpatients are presented in Equation (1), Equation (2), Equation (3), and Equation (4), respectively.

The overall probability density function using the F3 distribution for early outpatients is expressed by:

*f*(*x*) = [0.0178 × (−50.5 + 8 × BETA (1.120, 0.918))]   + [0.0537 × (−45.5 + 8 × BETA (1.440, 1.030))]   + [0.0440 × (−37.5 + 4 × BETA (1.050, 0.903))]    + [ 0.0438 × (−33.5 + 4 × BETA (1.540, 1.430))]     + [0.2007 × (−29.5 + 10 × BETA (1.130, 0.822))]     + [0.6400 × (−19.5 + 19 × BETA (0.981, 0.922))](1)

The overall probability density function using the F3 distribution for late outpatients is expressed by:

*f*(*x*) = [0.8327 × (0.5 + 12 × BETA (0.699, 1.390))] + [0.1673 × (12.5 + 8 × BETA (0.754, 1.060))] (2)

The overall probability density function using the F3 distribution for early inpatients is expressed by:

*f*(*x*) = [0.2770 × (−15.5 + 7 × BETA (1.560, 0.776))] + [0.7230× (−8.5 + 8 × BETA (1.020, 1.130))] (3)

The overall probability density function using the F3 distribution for late inpatients is expressed by:

*f*(*x*) = 0.5 + 10 × BETA (0.747, 1.530) (4)

### 3.4. Simulation Model Assumptions

The following list of assumptions was developed when constructing the simulation model:

- This study considered patients with a scheduled appointment at the case hospital. Only outpatients and inpatients were included. Emergent patients were excluded since they are considered walk-in patients.
- Patients may arrive unpunctually. Patients who arrived exactly at their appointment time were considered on-time patients. When a radiological technician is ready to serve the next ultrasound scan, the radiological technician will call the patient’s name (or patient’s appointment number). If the patient does not show up within 1 min, the radiological technician will call the next patient’s name. Therefore, patients who arrived 1 min earlier or 1 min later were considered unpunctual in this study.
- The case hospital’s scanning rooms for inpatients and outpatients operate Monday to Friday, from 9:00 am to 3:00 pm, with a 1-h break from 12:00 pm to 1:00 pm. Therefore, the scanning rooms operate 5 h per day for inpatients and outpatients.
- The case hospital has six rooms (rooms 5, 6, 7, 8, 9, and 10) that can provide any type of service to the patients. Six radiological technicians provide services. One radiological technician is assigned to each room. Eight types of services are provided to outpatients: Shoulder, scrotum, neck, prostate, thyroid, urotract, EXT DVT, and abdomen. Six types of services are provided to inpatients: EXT DVT, liver, prostate, urotract, thyroid, and abdomen.
- Patients’ walking times were not considered due to the adjacent locations between the check-in counter and scanning rooms. The probability distribution of the service time is given per body part based on the results of the data-fitting procedures.
- Patients were assumed to undergo the proper procedure when receiving the service.
- A constant arrival policy of 20 min was applied as the appointment scheduling policy used in the simulation system.

In determining the warm-up period used in the study, the simulation model operated for 12,000 min to determine the utilization rate of the six scanning rooms under steady-state conditions. When the simulation model reached 6000 min, no substantial fluctuations had occurred in the utilization rate. Therefore, the warm-up period applied in the simulation model was set to 6000 min.

Replication length was the total minutes per day multiplied by the number of simulation days plus the warm-up period in minutes, as shown in Equation (5). The total minutes per day was 300 min, and the number of simulation days was 5 days. Therefore, the replication length of the simulation model was 6000 + (300 × 5) = 7500 min.

Replication length = warm-up period + (minutes per day × number of days)(5)

In determining the number of replications in the study, Kelton et al.’s [42] method was applied. Equation (6) was used to calculate the required number of replications to be applied in the simulation model using the half-width value at the 95% confidence interval as follows:

(6) $$n\approx n_{0}\times \frac{h_{0}^{2}}{h^{2}}$$

where *n* was the required number of replications, *n*_0_ was the number of initial replications, *h* was the prespecified desired value of half-width, and *h*_0_ was the result of running the model under *n*_0_.

The number of replications was set to 30, and the total number of outpatients and inpatients entering the system was used to determine the required number of replications to be applied in the study. Table 5 shows the values of the average number of outpatients and inpatients ($\bar{x}$), the half-width (*h*) at the 95% confidence interval, and *h*/$\bar{x}$. As the values of *h*/$\bar{x}$ for both outpatients and inpatients were less than 1%, these values were within an acceptable range. Therefore, the number of replications was set to 30.

### 3.5. Verification and Validation of the Simulation Model

For the simulation model, the outpatients and inpatients were created and arrived at the hospital according to the F3 distribution applied, which represented the patient unpunctuality behavior. The percentages and known probability distribution of the scan time per body part assigned the patient to get their respective scan service. Thereafter, the patients were assigned to one of the six scanning rooms.

Regarding the logic of the simulation system, the simulation model would pass the verification test if it was ensured that the model behaved according to the researchers’ intention. After running the model, the researchers checked and agreed that the simulation model ran correspondingly. Therefore, the model was verified.

For simulation validation, this study was validated by comparing the actual average values to the simulated average values from the results of running the simulation model in a span of 5 days. This step ensured that the created simulation model behaved similarly or close to the actual system. Therefore, the model could be used to represent the real system and was good to be studied in the research. Using the half-width value at the 95% confidence interval, the allowable minimum and maximum range was acquired for use as an indicator that, if a given result lies within the range of the 95% confidence interval, it will have no significant difference, indicating that the result passed the validation [5,44,45].

Table 6 summarizes the actual and simulated average number of outpatients to be validated. As the focus of the study was to determine the impact of patient unpunctuality, the researchers divided the data according to patient behavior. For on-time and late outpatients, the average values of arriving outpatients were within the range of the 95% confidence interval of the simulated model. Only for early outpatients was the average value not in the range of the 95% confidence interval of the simulated model. For further analysis [46], the researchers referred to Lyu et al.’s [46] research. In their study, the average value of four performance indicators were also not in the range of the 95% confidence interval of the simulated model. The simulated-to-actual gap percentage of the four performance indicators was 10.97%, 13.44%, 9.87%, and 10.85%, respectively. The formula for the simulated-to-actual gap percentage is shown in Equation (7). Although the values of the simulated-to-actual gap percentage in their simulation model were close to or above 10%, their simulated model was still viewed as passing validation. In this case, the researchers calculated the simulated-to-actual gap percentage for the early outpatients. The simulated-to-actual gap percentage for the early outpatients was 2.48% (=|224.65−219.07|/224.65 × 100%). Due to the small simulated-to-actual gap percentage, the results for early outpatients were acceptable in this case. Therefore, the average number of early, on-time, and late outpatients entering the system was validated.

(7) $$\begin{array}{l} \mathrm{Simulated}-\mathrm{to}-\mathrm{actual}\;\mathrm{gap}\;\mathrm{percentage}\\ =\frac{\left|\mathrm{Actual}\;\mathrm{Average}\;-\;\mathrm{Simulated}\;\mathrm{Average}\right|}{\mathrm{Actual}\;\mathrm{Average}}\times 100\% \end{array}$$

Similarly, Table 7 summarizes the actual and simulated average number of inpatients to be validated. For early, on-time, and late inpatients, the average values of arriving inpatients were within the range of the 95% confidence interval of the simulated model. Therefore, the average number of early, on-time, and late inpatients entering the system was also validated.

The researchers also included the formulated F3 distribution in validating the simulation model to determine whether the F3 distribution could correctly represent the earliness and lateness behaviors of each patient. The summary of the values used for the validation is shown in Table 8 (outpatients) and Table 9 (inpatients). Only for the early inpatients was the average value of arriving inpatients not in the range of the 95% confidence interval of the simulated model. For these patients, the simulated-to-actual gap percentage calculated using Equation (7) was 2.80% (=|6.43−6.25|/6.43 × 100%). Due to the small simulated-to-actual gap percentage, the results for early inpatients were acceptable in this case.

### 3.6. What-If Scenarios

To determine the impact of patient unpunctuality on these two traditional policies (preempt and wait policies), the researchers tested different scenarios. To explain the scenario clearly, each scenario was considered using two different examples: The first with a constant service time and the second with a variable service time. The assumptions for the numerical examples are listed below:

1. The hospital opens at 9:00 am, at which point patients can wait inside for their appointment time.
2. The example hospital only has two rooms, which means that two patients are booked for each appointment slot.
3. The arrival time of each patient is independent.

#### 3.6.1. Preempt Policy

**Figure 2:**
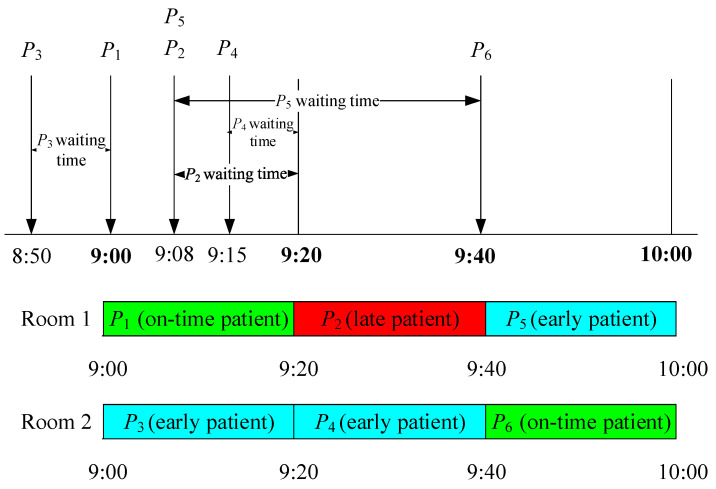
Examples of the preempt policy with constant service time.

**Figure 3:**
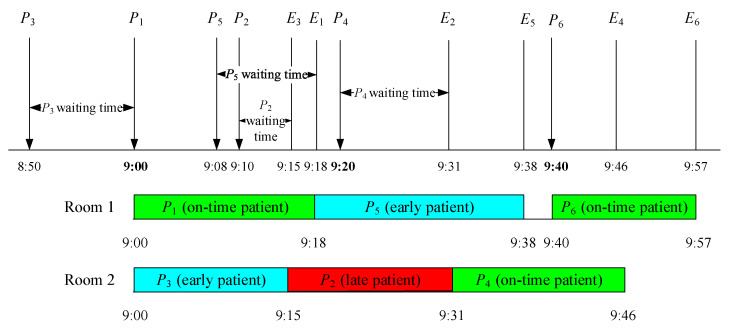
Examples of the preempt policy with variable service time.

Samorani and Ganguly [13] defined preemption as a service provider seeing the early patient as soon as the provider becomes idle. When a scheduled patient is not present at the scheduled time, service providers tend to preempt the available patient waiting in order to prevent idle time, which is the “always-preempt” policy [34]. However, this causes most patients to arrive earlier than their appointment time as they anticipate that they can get service immediately. To solve this dilemma, Cayirli et al. [14] suggested a first-scheduled-first-served policy. Preemption will be carried out, but the person to be preempted is decided based on the scheduled appointment order. On-time patients are always the priority. If a scheduled patient is present at the scheduled time, the patient will receive treatment immediately. Early and late patients will be queued and can be preempted based on their appointment schedule within the day. In this study, the researchers adapted Cayirli et al.’s [14] first-scheduled-first-served policy as the preempt policy. To further discuss the policy, two examples were considered: Constant service time (Figure 2 and Table 10) and variable service time (Figure 3 and Table 11).

#### 3.6.2. Examples of the Preempt Policy with Constant Service Time

For the constant service time example, all patients’ service time is assumed to be 20 min, and two scanning rooms are scheduled. For notations, *P_i_* is the arrival time of the *i*th patient, and *A_i_* is the scheduled appointment time of the *i*th patient. In Figure 2, *P*_3_, who has an appointment time at 9:20 am, arrives at 8:50 am; *P*_1_ then arrives at exactly 9:00 am (i.e., the scheduled appointment time), which means that *P*_1_ is on time. As *P*_2_ has not arrived yet and there is still one room available, *P*_3_ is preempted. At 9:08 am, *P*_2_ and *P*_5_ arrive; after 7 min, *P*_4_ arrives. Since the service starts at 9:20 am and P_4_’s scheduled appointment time is 9:20 am, *P*_4_ receives the service at the scheduled appointment time. *P*_2_ also receives the service at 9:20 am given the patient’s earlier scheduled appointment than *P*_5_, even though *P*_2_ is late and *P*_5_ is early. In this case, *P*_5_ receives the service at 9:40 am (i.e., the scheduled appointment time), as well as *P*_6_ since *P*_6_ is on time. The details of appointment time, arrival time, start of service time, end of service time, and waiting time for each patient are summarized in Table 11.

#### 3.6.3. Examples of the Preempt Policy with Variable Service Time

For the variable service time example, patients’ service time is assumed to be between 10 and 20 min, and two scanning rooms are scheduled. Except for *P_i_* and *A_i_*, *E_i_* is the end of the service time of the *i*th patient. Figure 3 shows the example for the preempt policy with variable service time wherein *P*_3_, an early patient, receives the service at 9:00 am since *P*_2_ has not arrived yet. At 9:08 am, *P*_5_, who has an appointment at 9:40 am, arrives, followed by the late patient *P*_2_ at 9:10 am. *P*_3_’s service ends at 9:15 am, meaning that the service provider is now available. Two patients, *P*_2_ and *P*_5_, are already in the queue. As the first-scheduled-first-served rule is being applied in this policy, *P*_2_ receives the service first even though *P*_5_ arrived first. *P*_5_ receives the service at 9:18 am, when *P*_1_’s service time ends. Since *P*_5_ has been preempted, there is no room available for the on-time patient *P*_4_. Therefore, *P*_4_ receives the service at the end of *P*_2_’s service, which occurs at 9:31 am. *P*_5_’s service ends at 9:38 am. Since *P*_6_ has not arrived yet, the service provider (i.e., Room 1) will be idle for 2 min. At 9:40 am, *P*_6_ will immediately receive the service. The details of appointment time, arrival time, start of service time, end of service time, and waiting time for each patient are summarized in Table 11.

Unpunctual patients will only be preempted if a scheduled patient missed the appointment time. The patient who will be preempted is based on who has the earliest scheduled appointment (first-scheduled-first-served policy). This policy was based on Cayirli et al.’s [14] paper, which issued the appointment schedule as the basis when preempting patients.

#### 3.6.4. Wait Policy

The wait policy also considers Deceuninck et al.’s [15] definition, where the appointment order is strictly followed. This means that the provider has to wait for the scheduled patient and remain idle until the scheduled patient arrives. Samorani and Ganguly [13] supported this type of policy by stating that, under some environmental parameters, waiting is beneficial. In this case, only early patients have to wait for their appointment schedule regardless of showing up earlier than the scheduled patient. However, for late patients, the researchers used one penalty policy developed in Klassen and Yoogalingam’s [11] paper. In their study, they tested four penalties: No penalty, late patients wait until the physician is idle, late patients are placed at the end of the existing queue, and late patients are placed at the end of the session. Among the four penalties, they found that late patients placed at the end of the existing queue outperformed the other policies. In this policy, on-time patients are still prioritized even if there is a patient in the waiting room. Therefore, in the current study, the researchers placed late patients at the end of the existing queue as the wait policy.

## 4. Results

### 4.1. Preempt and Wait Policies

Preempt and wait policies were carried out into the current model in order to determine which policy performs better with regard to patient unpunctuality. The researchers used the output analyzer in Arena Simulation Software to compare these two policies. The researchers computed the cost of patients’ waiting time and the radiological technicians’ idle time when dealing with patient unpunctuality. The formulas used to compute for the total cost were similar to the model of Zhu et al. [21]. Total cost was the main basis for comparing the policies, using the hypotheses and the 95% confidence interval.

H_0_: No significant difference exists between the mean of total cost of the preempt policy and the mean of total cost of the wait policy (*μ*_1_ − *μ*_2_ = 0).

H_1_: A significant difference exists between the mean of total cost of the preempt policy and the mean of total cost of the wait policy (*μ*_1_ − *μ*_2_ ≠ 0).

The results show that the null hypothesis (H_0_) at the 95% confidence interval was rejected. This means that a significant difference existed between the mean of total cost of the preempt policy and the wait policy. Since this difference (i.e., the mean of total cost of the preempt policy minus the mean of total cost of the wait policy) is negative, the total cost of the preempt policy is less than the total cost of the wait policy. With this, the preempt policy performs better with regard to the patient unpunctuality.

Both policies were able to serve all patients in 1 day. In the preempt policy, available early patients had a higher tendency to get service whenever the scheduled patient was not present at the scheduled time since early patients could be preempted, resulting in lower average waiting time and number of patients waiting compared to the wait policy, where early patients were forced to wait for their appointment time regardless of how early they entered the system. Idle time for the wait policy was higher than the preempt policy since it disregarded early patients even when the radiological technician and room were available but the scheduled patient was not present at that moment, resulting in more idle time. As the waiting time and idle time of the preempt policy were lower than those times of the wait policy, the waiting time cost and idle cost in the preempt policy would also be lower than the wait policy. Table 12 shows that the preempt policy indeed has lower costs than the wait policy. Please note that the cost of waiting for the patient is different from the radiological technician. The assumptions of (*ci*, *cw*) = (0.5, 0.5) do not expect these costs to be the same. They are the weightings between radiological technician’s idle time cost and patient waiting time cost in the system. The weightings are decided by the level of importance of the costs in the system.

These results support the study of Samorani and Ganguly [15]. According to their study, the preempt policy is the best policy for services with a short service time (i.e., 5–30 min) since it is easy to implement and has a small effect on waiting time; this finding can be applied in the current study as well, where the average service time of patients was 10 to 20 min.

### 4.2. Sensitivity Analyses

Sensitivity analyses were utilized on the preempt policy to see what happens to the system when key parameters were changed to different values. In this study, three key parameters are the patient’s inter-arrival time, cost coefficient of radiological technician’s idle time (*c_i_*), and cost coefficient of patient waiting time (*c_w_*), which were 20 min, 0.5, and 0.5, respectively.

We conducted four sensitivity analyses. First, the patient’s inter-arrival time was adjusted to ±2 min and ±4 min from the current value (i.e., 20 min). Using Zhu et al.’s [21] notations, the cost coefficients of radiological technician’s idle time and the patient waiting time were changed to give more weight to the latter cost coefficient since hospital managers focused on improving patient satisfaction. Therefore, the parameters of (*c_i_*, *c_w_*) were (0.4, 0.6), (0.3, 0.7), (0.2, 0.8), and (0.1, 0.9), while the base parameters were (0.5, 0.5).

#### 4.2.1. Base-Parameter Model Analysis

In Table 13, all patients (i.e., 54 patients) were served on the day of their appointment. The average number of patients waiting as well as the waiting time increased as the inter-arrival time decreased. The average total time in system and utilization of scanning rooms were also inversely proportional to the patient’s inter-arrival time. The number of early patients was lowest at the patient’s inter-arrival time of 24 min, while the number of late patients was lowest at 18 min. However, the number of on-time patients was highest at 22 min. No trend can be found regarding the number of early, late, and on-time patients and the patient’s inter-arrival time. Nonetheless, a patient’s inter-arrival time of 22 min would be preferable as patients tend to be more punctual. Furthermore, the utilization of scanning rooms was highest at a patient’s inter-arrival time of 18 min.

#### 4.2.2. Sensitivity Analysis 1

The cost coefficients of the radiological technician’s idle time and patient waiting time used in this sensitivity analysis were 0.4 and 0.6, respectively, for the five inter-arrival times. Table 14 shows the results for the first sensitivity analysis with the patient’s inter-arrival time (16, 18, 20, 22, and 24). When the patient’s inter-arrival time decreases (from 24, 22, 20, to 18), the radiological technician’s idle time cost increases. When the patient’s inter-arrival time decreases (from 22, 20, 18, to 16), the patient waiting time cost increases as well as the total cost. Although the radiological technician’s idle time cost with a patient’s inter-arrival time of 16 min is slightly less than a patient’s inter-arrival time of 18 min, the radiological technician’s idle time cost is inversely proportional to the patient’s inter-arrival time. Similarly, the patient waiting time cost and total cost are also inversely proportional to the patient’s inter-arrival time.

#### 4.2.3. Sensitivity Analysis 2

In this sensitivity analysis, the radiological technician’s idle time and the cost coefficients for patient waiting time used were 0.3 and 0.7, respectively, while considering five inter-arrival times. Table 15 shows the results for the second sensitivity analysis. The results for the three costs showed a similar trend as in the first sensitivity analysis. Therefore, the radiological technician’s idle time cost, the patient waiting time cost, and total cost are inversely proportional to the patient’s inter-arrival time.

#### 4.2.4. Sensitivity Analysis 3

In this sensitivity analysis, the cost coefficients for the radiological technician’s idle time and patient waiting time used were 0.2 and 0.8, while considering five inter-arrival times. Table 16 shows the results for the third sensitivity analysis. The results for the radiological technician’s idle time cost, the patient waiting time cost, and total cost showed a similar trend as in the first and second sensitivity analyses. Therefore, the radiological technician’s idle time cost, the patient waiting time cost, and total cost are inversely proportional to the patient’s inter-arrival time.

#### 4.2.5. Sensitivity Analysis 4

This sensitivity analysis sets the cost coefficients for radiological technician’s idle time and patient waiting time to 0.1 and 0.9, while considering five inter-arrival times. Table 17 shows the results for the third sensitivity analysis. The results for the radiological technician’s idle time cost, the patient waiting time cost, and total cost showed a similar trend as in the first and second sensitivity analyses. Therefore, the radiological technician’s idle time cost, the patient waiting time cost, and total cost are inversely proportional to the patient’s inter-arrival time.

## 5. Discussion

According to the four sensitivity analyses, a 24-min patient’s inter-arrival time resulted in the lowest cost for the radiological technician’s idle time since the average total time in system of a patient is 26.17 min, which is close to the 24-min interval. The approximate 2.17-min difference between the interval and total time in system means that the radiological technician remains engaged in work and has less idle time. In addition, a 22-min patient’s inter-arrival time resulted in the lowest cost for patient waiting time. Service time for each patient usually took approximately 19 to 20 min, meaning that patients had an extra 2 min before arriving for their scheduled appointment, during which the radiological technician could prepare for the next appointment. An inter-arrival time of 16 or 18 min might be preferred to 22 min to ensure that the patient arrives before the previous patient finishes the service. However, this approach would cause congestion in the waiting room, making it more crowded and resulting in patient dissatisfaction [13,47].

As the patient’s inter-arrival decreased, the radiological technician’s idle time increased, resulting in a higher idle cost, which greatly affects the total cost as well as the cost coefficient of patient waiting time. The average number of patients waiting only differs when the patient’s inter-arrival time is adjusted, yet it remains the same throughout all cost coefficients since it does not deal with costs. It is apparent that the number of patients waiting is higher when the patient’s inter-arrival interval is shorter than when it is long. The same is true with the patients’ average waiting time. Patients incur more waiting time when the patient’s inter-arrival interval is shorter than when it is long. As previously mentioned, this result is mainly due to the service time usually taking about 19 to 20 min. However, if the patient’s inter-arrival is set lower than the service time, patients will arrive on time while the radiological technician is still working with the prior patient, meaning that the technician is not ready to provide service to the scheduled patient. When the patient’s inter-arrival time increases, utilization decreases. When the patient’s inter-arrival was 24 min, utilization decreased slightly, mainly due to idle time. As service usually takes about 19 to 20 min, when the patient’s inter-arrival is 24 min, the 4- to 5-min difference will lead to idle time and low utilization of the scanning rooms.

In the four sensitivity analyses, the results showed that different settings of patient’s inter-arrival have an impact on the three costs (i.e., radiological technician’s idle time cost, the patient waiting time cost, and total cost); however, different settings of cost coefficients for radiological technician’s idle time and patient waiting time did not have an impact on the three costs. Therefore, the key parameter was the patient’s inter-arrival for patient unpunctuality.

Unpunctuality has numerous impacts on the system. All patients are served within the scheduled day, but they may face waiting and idle times. The waiting and idle times depend on the patient’s inter-arrival time that is set in the schedule policy and the degree of patients’ unpunctuality. The sensitivity analyses demonstrated how the system works in the presence of patient unpunctuality and different scenarios.

## 6. Conclusions

By comparing the total cost of each policy, the researchers determined that the preempt policy is better than the wait policy as the total cost of the former is less than the total cost of the latter. Therefore, the preempt policy performs better than the wait policy in the presence of patient unpunctuality. These findings support those of Samorani and Ganguly [15], who concluded that the preempt policy is the best policy for services with a short service time since it is easy to implement and has a small effect on the waiting time.

In the presence of patient unpunctuality, the weights of the cost coefficients of both radiological technician’s idle time and patient waiting time must be equal in order to achieve lower costs. The patient’s inter-arrival time must be close to the average total time in the system to ensure lower costs. To prevent patient waiting time and congestion in the waiting room, the patient’s inter-arrival time should be higher than the service time, but should still be close to it. Moreover, utilization decreases as the patient’s inter-arrival increases. Therefore, patient’s inter-arrival time should be higher than, but near to, the service time to ensure lower patient waiting time and radiological technician’s idle time.

The limitations of this study included two factors. First, this research assumed that there is neither walk-ins (i.e., emergency patients) nor no-show patients for the case study. However, walk-ins and no-show patients exist for patient appointment scheduling systems. No-show patients for one-doctor clinics have more impact on patient appointment scheduling systems than those for multi-doctor ones. How to integrate walk-ins and no-show patients with patient unpunctuality into patient appointment scheduling problems merits further research. Second, this research used the one-patient in one-time-slot policy with patient unpunctuality for patient appointment schedules. Although patient service time can be constant or variable, different patient appointment scheduling policies with patient unpunctuality also merit further research. Therefore, two future research directions should be explored. First, researchers could incorporate no-show patients or walk-ins into patient appointment scheduling problems with patient unpunctuality to investigate the impact on system performance [11,12,26]. Second, researchers could explore more policies for patient appointment scheduling problems with patient unpunctuality in order to determine a better patient appointment scheduling policy [6,48,49].

## Figures and Tables

**Table 1:** F3 distribution for early outpatients.

Range of Patient Waiting Time (Minutes)		Percentage (%)	Cumulative Percentage (%)	Probability Distribution
Lower Limit	Upper Limit			
−50	−46	1.78	1.78	−50.5 + 8 × BETA (1.120, 0.918)
−45	−36	5.37	7.15	−45.5 + 8 × BETA (1.440, 1.030)
−37	−34	4.40	11.55	−37.5 + 4 × BETA (1.050, 0.903)
−33	−30	4.38	15.93	−33.5 + 4 × BETA (1.540, 1.430)
−29	−20	20.07	36.00	−29.5 + 10 × BETA (1.130, 0.822)
−19	−1	64.00	100.00	−19.5 + 19 × BETA (0.981, 0.922)

**Table 2:** F3 distribution for late outpatients.

Range of Patient Waiting Time (Minutes)		Percentage (%)	Cumulative Percentage (%)	Probability Distribution
Lower Limit	Upper Limit			
1	12	83.27	83.27	0.5 + 12 × BETA (0.699, 1.390)
13	20	16.73	100.00	12.5 + 8 × BETA (0.754, 1.060)

**Table 3:** F3 distribution for early inpatients.

Range of Patient Waiting Time (Minutes)		Percentage (%)	Cumulative Percentage (%)	Probability Distribution
Lower Limit	Upper Limit			
−15	−9	27.70	27.70	−15.5 + 7 × BETA (1.560, 0.776)
−8	−1	72.30	100.00	−8.5 + 8 × BETA (1.020, 1.130)

**Table 4:** Probability distribution for late inpatients.

Range of Patient Waiting Time (Minutes)		Percentage (%)	Cumulative Percentage (%)	Probability Distribution
Lower Limit	Upper Limit			
1	10	100.00	100.00	0.5 + 10 × BETA (0.747, 1.530)

**Table 5:** Number of patients entering the system in 5 days after 30 replications.

Patient Type	$\mathrm{Average}\;\mathrm{Number}\;\mathrm{of}\;\mathrm{Patients}\;(\bar{x})$	Half-Width at 95% Confidence Interval (*h*)	$\mathrm{Half}-\mathrm{Width}/\mathrm{Average}\;\mathrm{Number}\;\mathrm{of}\;\mathrm{Patients}\;(h/\bar{x})$ in %
Outpatients	262.63	0.46	0.18
Inpatients	50.07	0.28	0.56

**Table 6:** Outpatient actual average number of arrivals and simulated average number of arrivals in 5 days.

Patient Type	Actual Average Number of Patients	Simulated Average Number of Patients	Range at 95% Confidence Interval of Simulated Model	
			Minimum	Maximum
Early Outpatients	224.65 *	219.07	216.52	221.62
On-time Outpatients	8.22	8.23	7.05	9.41
Late Outpatients	41.01	38.70	36.28	41.12

* A simulated-to-actual gap percentage computation is needed to support this validation.

**Table 7:** Inpatient actual average number of arrivals and simulated average number of arrivals in 5 days.

Patient Type	Actual Average Number of Patients	Simulated Average Number of Patients	Range at 95% Confidence Interval of Simulated Model	
			Minimum	Minimum
Early Inpatients	38.33	39.37	38.09	40.65
On-time Inpatients	2.33	2.53	1.92	3.14
Late Inpatients	9.30	9.10	7.90	10.30

**Table 8:** Actual and simulated average number of outpatient arrivals based on probability distribution.

Patient Type	Actual Average Number of Patients	Simulated Average Number of Patients	Range at 95% Confidence Interval	
			Minimum	Minimum
Early Outpatients	17.02	17.06	16.89	17.23
Late Outpatients	6.50	6.60	6.33	6.87

**Table 9:** Actual and simulated average number of inpatient arrivals based on probability distribution.

Patient Type	Actual Average Number of Patients	Simulated Average Number of Patients	Range at 95% Confidence Interval	
			Minimum	Minimum
Early Inpatients	6.43 *	6.25	6.10	6.40
Late Inpatients	3.79	3.66	2.87	4.45

* A simulated-to-actual gap percentage computation is needed to support this validation.

**Table 10:** Preempt policy with constant service time.

Patient	Appointment Time	Arrival Time	Early, Late, or On-Time	Start of Service Time	End of Service Time	Waiting Time (Minutes)	Room No.
*P*1	9:00	9:00	On-time	9:00	9:20	0	1
*P*3	9:20	8:50	Early	9:00	9:20	10	2
*P*2	9:00	9:08	Late	9:20	9:40	12	1
*P*4	9:20	9:15	Early	9:20	9:40	5	2
*P*5	9:40	9:08	Early	9:40	10:00	32	1
*P*6	9:40	9:40	On-time	9:40	10:00	0	2

**Table 11:** Preempt policy with variable service time.

Patient	Appointment Time	Arrival Time	Early, Late, or On-Time	Start of Service Time	End of Service Time	Waiting Time (Minutes)	Room No.
*P*_1_	9:00	9:00	On-time	9:00	9:18	0	1
*P*_3_	9:20	8:50	Early	9:00	9:15	10	2
*P*_2_	9:00	9:10	Late	9:15	9:31	5	2
*P*_5_	9:40	9:08	Early	9:18	9:38	10	1
*P*_4_	9:20	9:20	On-time	9:31	9:46	11	2
*P*_6_	9:40	9:40	On-time	9:40	9:57	0	1

**Table 12:** Cost analysis of the preempt policy and the wait policy.

Cost	Preempt Policy	Wait Policy
Radiological Technician’s Idle Time Cost	NT 5809.97	NT 5872.74
Patient Waiting Time Cost	NT 888.87	NT 916.95
Total Cost	NT 3349.41	NT 3394.85

Note: Total Cost = 0.5 × Radiological Technician’s Idle Time Cost + 0.5 × Patient Waiting Time Cost. Here, (*c_i_*, *c_w_*) = (0.5, 0.5). Here, NT represent New Taiwan dollars.

**Table 13:** Base-parameter analysis with the pair cost coefficients (0.5, 0.5).

Key Performance Index	Patient’s Inter-Arrival Time (Minutes)				
	16	18	20	22	24
Radiological Technician’s Idle Time Cost (NT Dollars)	5762.35	5863.52	5809.94	5718.61	5716.29
Patient Waiting Time Cost (NT Dollars)	1623.80	1352.32	888.87	817.28	826.05
Total Cost (NT Dollars)	3693.08	3607.92	3349.41	3267.95	3271.17
No. of Patients Served (Patients)	54	54	54	54	54
Average Number of Patients Waiting (Patients)	0.99	0.84	0.54	0.49	0.49
No. of Early patients (Patients)	44.27	45.07	44.80	44.40	44.13
No. of Late patients (Patients)	8.07	7.20	7.67	7.80	8.13
No. of On-time patients (Patients)	1.66	1.73	1.53	1.80	1.74
Average Waiting Time (Minutes)	12.89	10.73	7.05	6.49	6.56
Average total Time in System (Minutes)	32.93	30.75	27.13	26.33	26.17
Utilization of Scanning Rooms (%)	53.72	53.86	53.72	53.55	52.58

**Table 14:** Sensitivity analysis 1 with the pair cost coefficients (0.4, 0.6).

Key Performance Index	Patient’s Inter-Arrival Time (Minutes)				
	16	18	20	22	24
Radiological Technician’s Idle Time Cost (NT Dollars)	3457.41	3518.11	3485.98	3431.17	3429.77
Patient Waiting Time Cost (NT Dollars)	649.52	540.93	355.55	326.91	330.42
Total Cost (NT Dollars)	1772.68	1731.80	1607.72	1568.61	1570.16

**Table 15:** Sensitivity analysis 2 with the pair cost coefficients (0.3, 0.7).

Key Performance Index	Patient’s Inter-Arrival Time (Minutes)				
	16	18	20	22	24
Radiological Technician’s Idle Time Cost (NT Dollars)	4033.65	4104.46	4066.98	4003.03	4001.40
Patient Waiting Time Cost (NT Dollars)	487.14	405.70	266.66	245.18	247.82
Total Cost (NT Dollars)	1551.09	1515.33	1406.76	1372.54	1373.89

**Table 16:** Sensitivity analysis 3 with the pair cost coefficients (0.2, 0.8).

Key Performance Index	Patient’s Inter-Arrival Time (Minutes)				
	16	18	20	22	24
Radiological Technician’s Idle Time Cost (NT Dollars)	4609.88	4690.82	4647.98	4574.89	4573.03
Patient Waiting Time Cost (NT Dollars)	324.76	270.46	177.77	163.46	165.21
Total Cost (NT Dollars)	1181.78	1154.53	1071.81	1045.75	1046.77

**Table 17:** Sensitivity analysis 4 with the pair cost coefficients (0.1, 0.9).

Key Performance Index	Patient’s Inter-Arrival Time (Minutes)				
	16	18	20	22	24
Radiological Technician’s Idle Time Cost (NT Dollars)	5186.12	5277.17	5228.97	5146.75	5144.66
Patient Waiting Time Cost (NT Dollars)	162.38	135.23	88.89	81.73	82.61
Total Cost (NT Dollars)	664.75	649.42	602.90	588.23	588.82

## Data Availability

Not applicable.
